# Metal–organic framework derived nanomaterials for electrocatalysis: recent developments for CO_2_ and N_2_ reduction

**DOI:** 10.1186/s40580-020-00251-6

**Published:** 2021-01-05

**Authors:** Chanderpratap Singh, Subhabrata Mukhopadhyay, Idan Hod

**Affiliations:** Department of Chemistry and Ilse, Katz Institute for Nanoscale Science and Technology, Ben- Gurion University of Negev, 8410501 Beer-Sheva, Israel

**Keywords:** Metal–organic fameworks (MOFs), MOF-derived materials, Electrocatalysis

## Abstract

In recent years, we are witnessing a substantially growing scientific interest in MOFs and their derived materials in the field of electrocatalysis. MOFs acting as a self-sacrificing template offer various advantages for the synthesis of carbon-rich materials, metal oxides, and metal nanostructures containing graphitic carbon-based materials benefiting from the high surface area, porous structure, and abundance of metal sites and organic functionalities. Yet, despite recent advancement in the field of MOF-derived materials, there are still several significant challenges that should be overcomed, to obtain better control and understanding on the factors determining their chemical, structural and catalytic nature. In this minireview, we will discuss recently reported advances in the development of promising methods and strategies for the construction of functional MOF-derived materials and their application as highly-active electrocatalysts for two important energy-related reactions: nitrogen reduction to produce ammonia, and CO_2_ reduction into carbon-based fuels. Moreover, a discussion containing assessments and remarks on the possible future developments of MOF-derived materials toward efficient electrocatalysis is included.

## Introduction

In modern society, there is a constantly rising demand for the supply of energy sources, in order to sustain the global economy growth. Nowadays, fossil fuels based on hydrocarbons such as petroleum, natural gas, and coals act as the main energy sources to fulfill this vast energy demand [1, 2]. As an outcome, there is an alarming growth in carbon-emissions, which are directly affects global warming. The rapid depletion of fossil fuels opens up a major quest for the scientific community to find alternative sources of fuels. In that regard, electrocatalysis is considered as one of the most promising technologies that could replace the currently used fossil fuels, and be utilized for the production of fine-chemicals and fuels in a clean, cheap and efficient manner [3–6].

Nevertheless, in order to achieve this ambitious goal, one need to design and develop suitable functional materials that can effectively catalyze the desired reactions. in that context, various porous materials have been explored for this purpose, such as porous carbons, Metal-Organic Frameworks (MOFs), metal/metal-oxide nanoparticles [7–15]. Moreover, porous carbon-based materials and their composites with metal-oxide nanoparticles are viewed as important catalytic materials due to their excellent electrochemical activity, high chemical and thermal stability, and the possibility for their rational design according to the requirement of a specific application [16, 17].

**Fig. 1:**
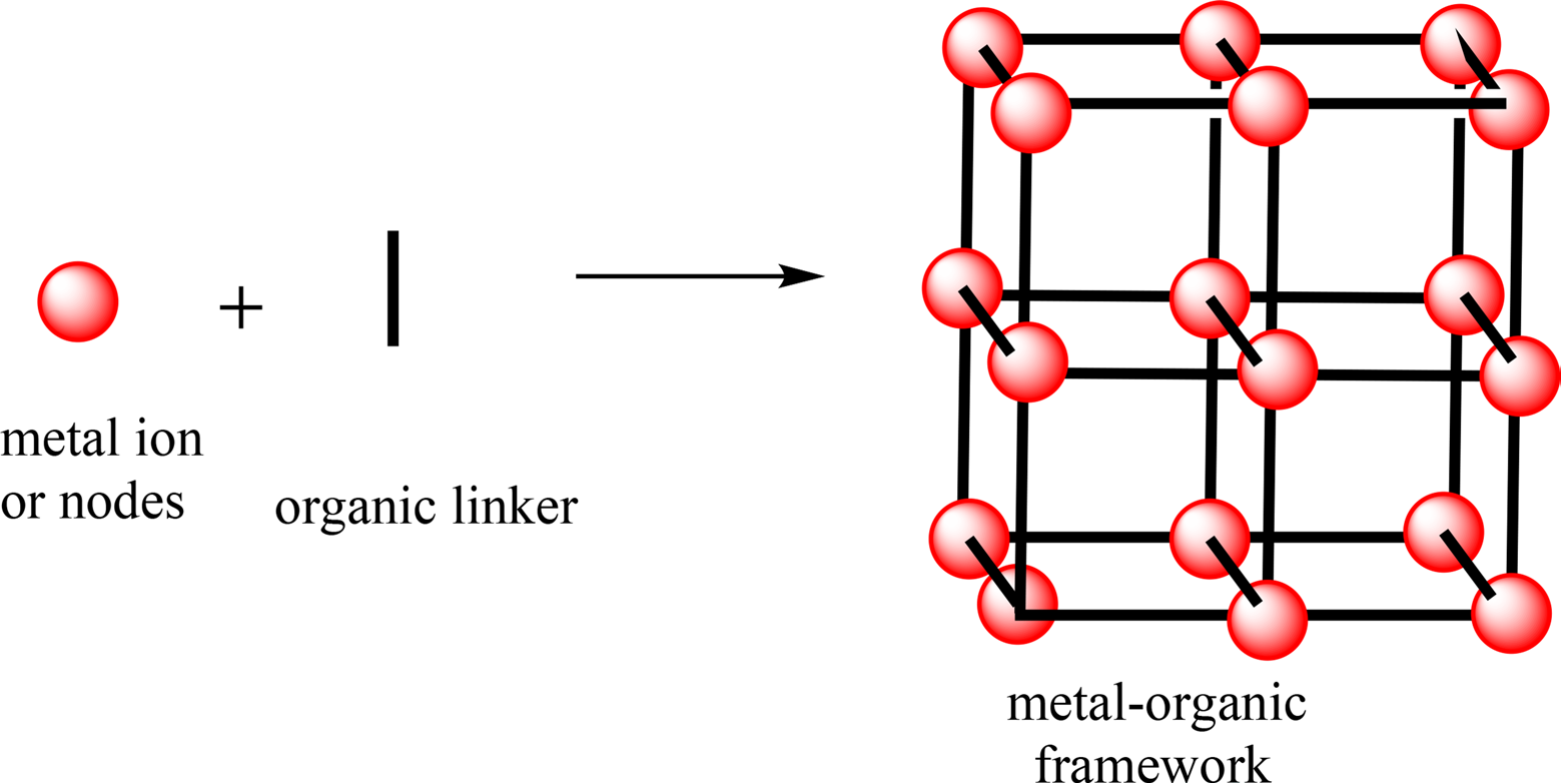
Illustration of MOF’s building blocks and resulting porous, crystalline structure.

MOFs, a family of crystalline porous coordination polymers, are built from repeating structural units of metal ions/clusters and multi-topic organic ligands (see illustration in Fig. 1) [3, 6, 18]. These materials have MOFs The unique features of MOFs such as high surface area, tunable porosity, design flexibility, and ease of chemical-functionalization [19] provide major advantage over other existing materials. As an outcome of these mentioned virtues, over the passed two decades, MOFs have attracted great attention from the scientific community due to the scope of using either pristine MOFs or MOF-derived materials in various important applications. They have also been extensively evaluated as highly promising candidate materials for the design of efficient renewable energy based electrochemical devices e.g., supercapacitors, batteries, and electrocatalysis [5, 17, 20–23].

To date, pristine MOF-based systems have been studied in various vital electrocatalytic processes e.g., oxygen evolution reaction (OER), oxygen reduction reaction (ORR), hydrogen evolution reaction (HER), CO_2_ reduction reaction (CO_2_RR), and N_2_ reduction reaction (NRR). However, despite the the progress made in recent years, pristine MOFs electrocatalysts are often found to exhibit inferior performance compared to conventional, heterogeneous inorganic electrocatalysts, mainely due to: (i) their intrinsicly low electrical conductivity, (ii) moderate stability under the conditions of electrochemical performance.

Alternatively, in order to overcome the aforementioned drawbacks of pristine MOFs, one can use them as a sacrificial soft and hard templates, to fabricate various conducting carbon-based nanomaterials such as porous graphitic carbon materials, N-rich graphitic carbons, heteroatom doped carbon materials, and metal/metal-oxide doped carbons. These MOF-derived materials offer tailorable morphologies, hierarchical porous structures (exposing high density of catalytically-active sites), ease of surface-functionalization, heteroatom doping, and also superior electrical conductivity, ionic conductivity, and chemical/structural stability compared to their pristine MOF precursors [13, 24, 25].

**Fig. 2:**
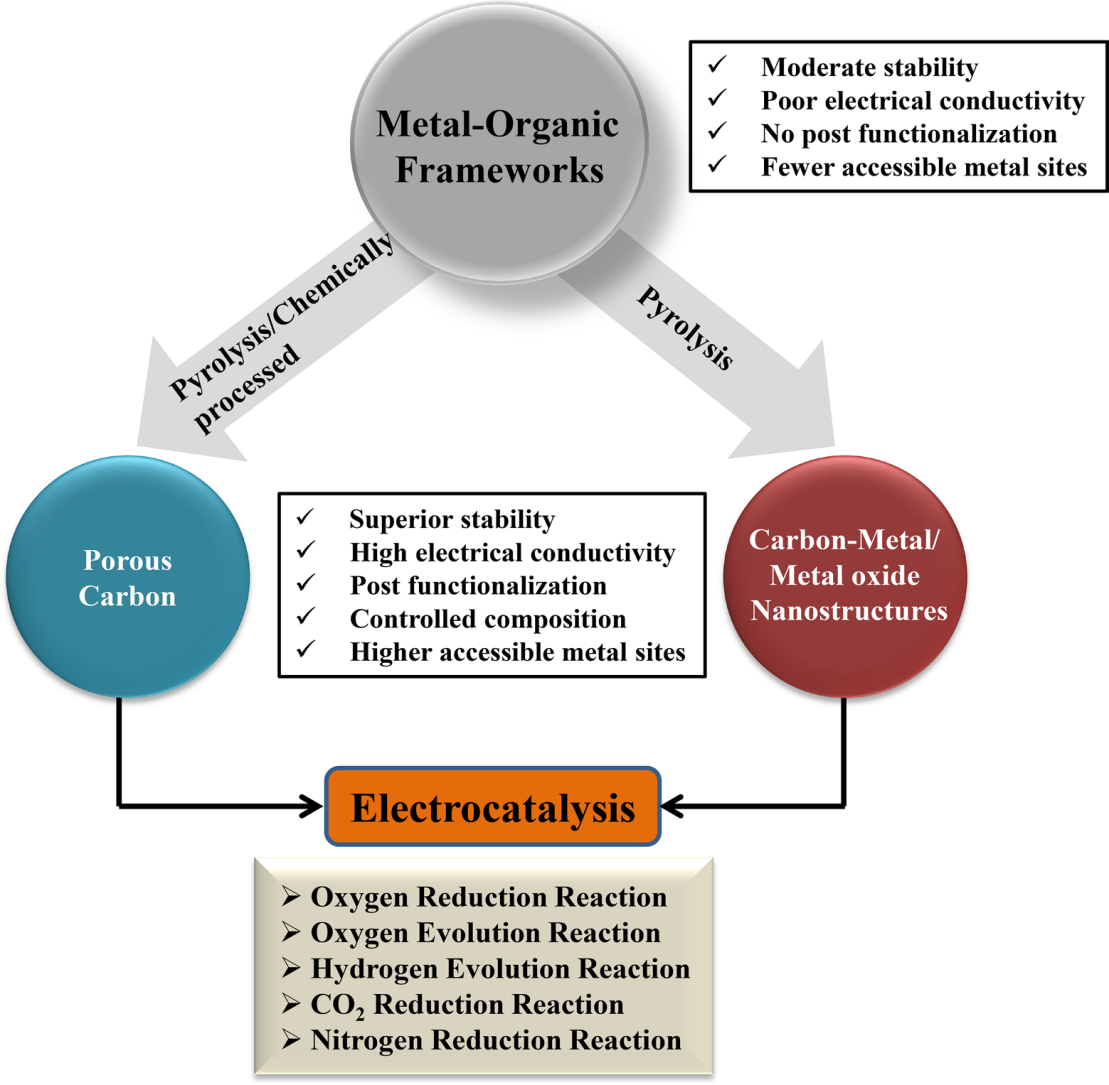
Summary of the different routes for the synthesis of MOF-derived porous graphitic carbon-rich materials, alongside their chemical, electrical and catalytic properties and application in electrocatalysis

Generally, the following strategies have been followed widely for the synthesis of MOF-derived materials: (1) direct MOF pyrolysis; (2) chemical decomposition; (3) pyrolysis of precursor-mixtures containing MOFs and added various substances [9, 26, 27]. Depending on the reaction condition, the thus formed derivatives cover a wide range of chemical composition ranging from metal-oxides, carbides, nitrides, sulfides and phosphides [25, 28–31]. Additionally, various carbon-based materials could be formed, having great potential in the fields of energy conversion and storage. The following approaches have been adopted widely for controlling the chemical composition of MOF-derived material: (1) by tuning the composition of MOF precursor; (2) controlled tuning of MOF derived structure during the conversion process; (3) post-synthesis modification [32–34]. Figure 2, depicts a typical approach to design MOF-derived materials. On account of these benefits, MOF-derived materials have been reviewed by many researchers in the field of electro-catalysis. This review will account for the recent developments of the synthesis and electrocatalytic applications of various MOF-derived materials (as summarized in Fig. 2). In addition, an emphasis will be given to 2 key and highly explored catalytic conversion reactions: nitrogen reduction to ammonia and CO_2_ reduction to sustainable fuels. Finally, possible future perspectives for further advancing the field of MOF-derived materials will be discussed. We believe that this review will aid readers in finding new methods for the synthesis of MOF-derived materials while pointing for different manner in which they could be utilized to achieve efficient electrocatalysis.

## MOF-derived electrocatalysts

Most of the crucial electrochemical processes required for the generation of clean, renewable energy sources require the application of high overpotentials in order to perform the process at a practical catalytic rate e.g., oxygen reduction (ORR), oxygen evolution reaction (OER), hydrogen evolution reaction (HER), which are key reactions of fuel-cells and water splitting applications. Thus, there is an urgent need to develop efficient and stable electrocatalyst to carry out these processes. With increasing alarming effects of global warming, reduction of CO_2_ to useful chemicals, and conversion of atmospherically abundant N_2_ to ammonia can be of utmost importance in the near future. Electrocatalysts can dramatically reduce the activation energy barrier for the conversion process and hence accelerate both the reaction rates as well as the efficiency of chemical transformations. Noble transition-metal-based electrocatalysts are often found to be very efficient, yet are not suitable for large scale applications as they are highly expensive. Thus, there is an ongoing demand for the design and development of new, efficient, robust and cost-effective electrocatalysts constructed with highly abundant elements. In that regard, over the last years, MOF-derived materials constitute one of the most promising candidates for alternative electrocatalytic compounds, as will be further discussed in the next sections of this minireview. As illustrated in Fig. 2, depending on the synthetic conditions of MOF conversion, one can obtain either nanostructured porous carbons, or carbon-metal/metal-oxide composites.

## Porous carbon nanostructures

**Fig. 3:**
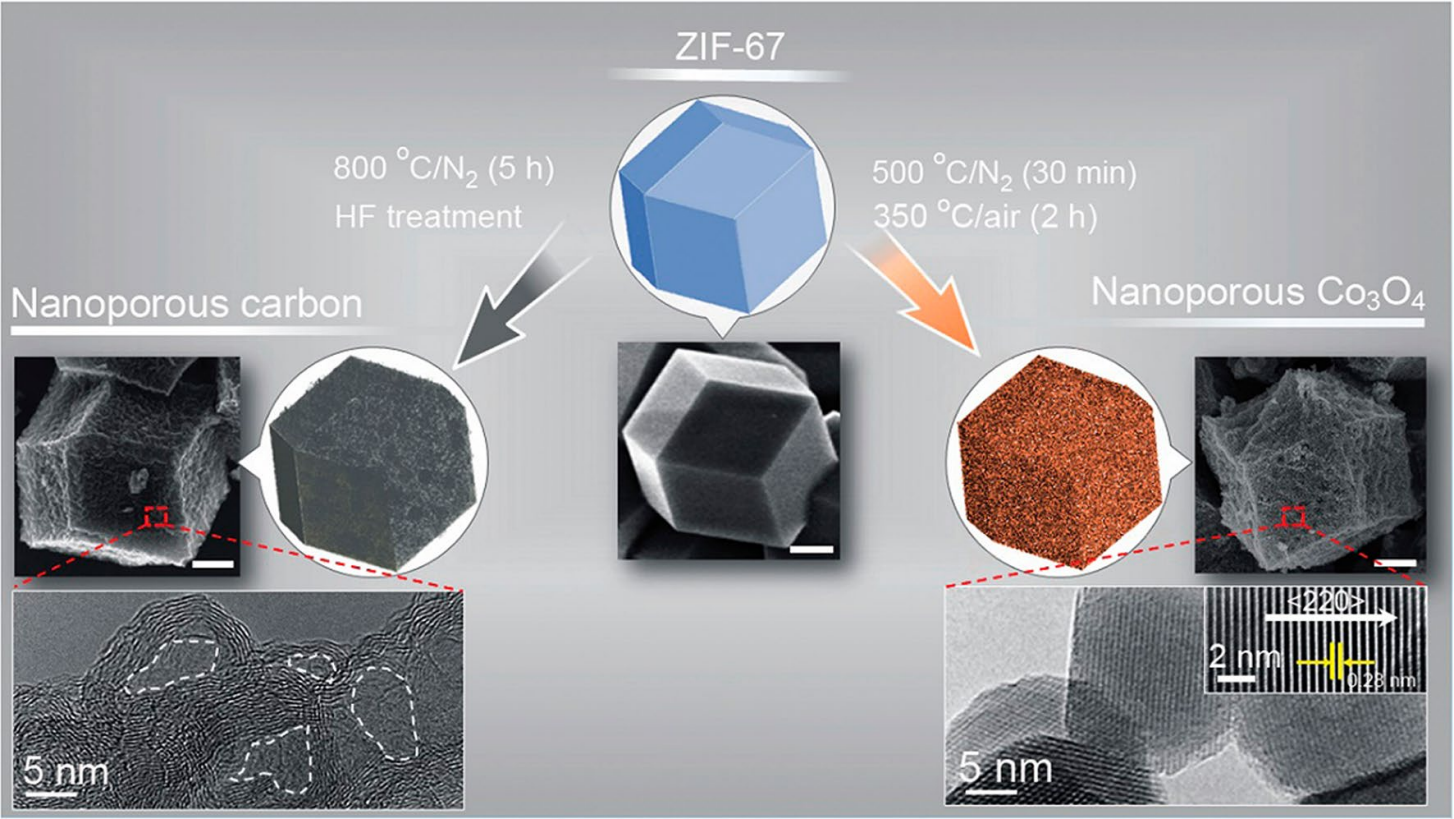
Schematic representation of the preparation of nanoporous carbon and nanoporous Co_3_O_4_ from a ZIF-67 polyhedron with their respective SEM and high-resolution TEM images. The scale bar for SEM image is 500 nm in length. TEM analysis suggests the removal of cobalt nanoparticles from the nanoporous carbon surface after thermal treatment and HF washing (as marked by the white dotted lines). However in the case of Co_3_O_4_, the optimized heating conditions result in the conversion of the ZIF-67 polyhedron into an oxide polyhedron. Reproduced with permission from Ref. [20]. Copyright 2015, American Chemical Society

Electrically-conducting porous carbon materials offer excellent chemical, electrical, thermal, and surface properties, with distinct morphologies and dimensionalities, ranging from zero-dimensional (0D) to three dimensional (3D) carbon networks [35, 36]. Typically, MOFs acts as an ideal precursor material for the synthesis of such porous carbons, as they provide the ability to gain fine control over the end-product’s crystallinity and degree of graphitization, as a result of their well-defined structural properties and distinctive decomposition features during high-temperature pyrolysis conditions [21, 37–39]. For pyrolysis-based MOF-derived synthesis of nanostructured porous carbons, one can distinguish between several experimental parameters, namely the pyrolysis temperature, the composition of reaction’s gas/gas mixture, and post-synthetic chemical treatments [40, 41]. Depending upon the used temperature and reaction environment, it is possible to control the degree of graphitization (and hence tune the surface area and electronic properties) of the carbonaceous product. Upon thermal treatment, the obtained metal nanostructures embedded in the carbon matrix can be removed by an acid wash step followed by and high-temperature evaporation [21, 24]. Salunkhe et al. reported on a ZIF-67 derived carbon to posses graphitized carbon-containing walls with a high specific surface area of 350 m^2^ g^− 1^ under N_2_ atmosphere at 800 °C, followed by HF treatment as shown in Fig. 3, to remove the metal ions [20]. Several notable examples of MOF-derived carbonaceous materials include: (i) Xia et al. reported the synthesis of N-doped carbon nanotubes using direct thermal treatment of ZIF-67 particles under Ar/H_2_ atmosphere at 700 °C  [34]. (ii) Ye et al. fabricated 3D porous carbons by the pyrolysis of oxygen-rich zinc-containing MOF [42]. (iii) Zhang et al.. invented highly graphitized nitrogen-doped porous carbon with a very high specific surface area of 932 m^2^ g^−1^ using ZIF-8, under N_2_ atmosphere at 1000 °C for 10 h [39]. (iv) Liu et al. synthesized double-shelled hybrid carbon nanocage derived from the epitaxial grown ZIF-67 crystals over ZIF-8 (ZIF-8@ZIF-67). ZIF-8@ZIF-67 were subjected to carbonization at 700 °C under N_2_ atmosphere followed by acid washing results unique double-shelled hybrid carbon nanocage with high specific surface area of 467 m^2^ g^− 1 ^[43]. All These aforementioned carbon-based nanomaterials exhibits desired virtues for an excellent electrocatalyst:high specific surface area, rexcellent electrical conductivity and mass-transport properties, structural robustness coupled with remarkable chemical stability over a wide range of pH, which makes them potential candidates for various energy related electrocatalytic applications [42].

## Carbon–metal/metal-oxide nanostructures derived via MOF pyrolysis

Generally, carbon-metal nanostructures can be synthesized by direct decomposition of MOFs under controlled temperature and a suitable environment as mentioned before. As a result of the thermal treatment, the organic linkers decompose causing the framework structure to break down. In the process, it creates high porosity in the matrix during the removal of the volatile molecules and leaves behind metal nanoparticles/ metal oxide nanoparticles embedded within highly electrically conducting carbon matrix. The embedded metal nanoparticles on the porous carbon matrix offer features like excellent electrical conductivity, high density of catalytically-active sites, while preserving the ability to accommodate solution-based mass-transport, an essential aspect to allow efficient electrocatalysis. There are various pathways to produce carbon-metal nanostructured composites. It is important to develop control over crystallinity, graphitization and composition of the MOF-derived carbon-metal electrocatalysts, which can be achieved varying metal centre (mono/bi-metallic), decomposition temperature, and post-synthesis modifications [32, 44]. For instance, as highlighted by Zhang et al. bi-metallic MOF-based precursors were synthesized and subsequently were subjected to pyrolysis treatment result improved oxygen-reduction performance in polymer-electrolyte-membrane fuel cells [45]. The decomposition temperature plays a major rule indefining the end product’s electrical nature. As a rule of thumb, it has been widely observed that during pyrolysis, the greater the temperature of thermal annealing under an inert atmosphere, the greater is the scope of graphitization [46]. Singh et al. have grown Fe/Ni-MIL-53 over carbon cloth *via* electrophoretic deposition and pyrolyzed at a different temperature to achieve metal embedded carbons [47]. Kampouri et al. synthesized mixed face TiO_2_ embedded in a carbon framework, by pyrolyzing a Ti-based MIL-125-NH_2_ at various temperatures [48]. Using post-synthesis modification strategy Chen et al. have successfully anchored W atoms on N-doped carbon matrix derived from the mixing of WCl5 and UiO-66-NH2 at an elevated temperature 900 °C [24]. Shin et al. produced porous carbon decorated with inexpensive metals nanomaterial Cu and Co (Cu, CO@PC) derived from ZIF-67 at 700 °C under N_2_ atmosphere [49]. Hu et al. have developed a new strategy to obtain ternary composites of Co/CoN/CoP_2_ out of a Co-MOF precursor [26]. To attain the different composites, the MOF powder was subjected to carbonization by mixing it with different sources of nitrogen and boron at an elevated temperature of 600 °C. Additionally, Zhang et al. have used a multi-step strategy to derive a Co@BCN based core-shell catalyst from ZIF-67 [37]. To do so, the ZIF-67 starting material was subjected to carbonization with different sources of boron and nitrogen to receive N and B doped carbon cages Co@BCN, Co@CN, and Co@C. Luo et al. synthesized Co_3_O_4_ based nanocomposites with N-doped carbon from a ZIF-67 precursor, by multi-step oxidation and reduction [38]. Salunkhe et al.. reported ZIF-67-derived nanoporous Co_3_O_4_ using a two-step thermal treatment, under N_2_ and ambient atmosphere at 500 °C, and 350 °C respectively (Fig. 3) [20].

**Fig. 4:**
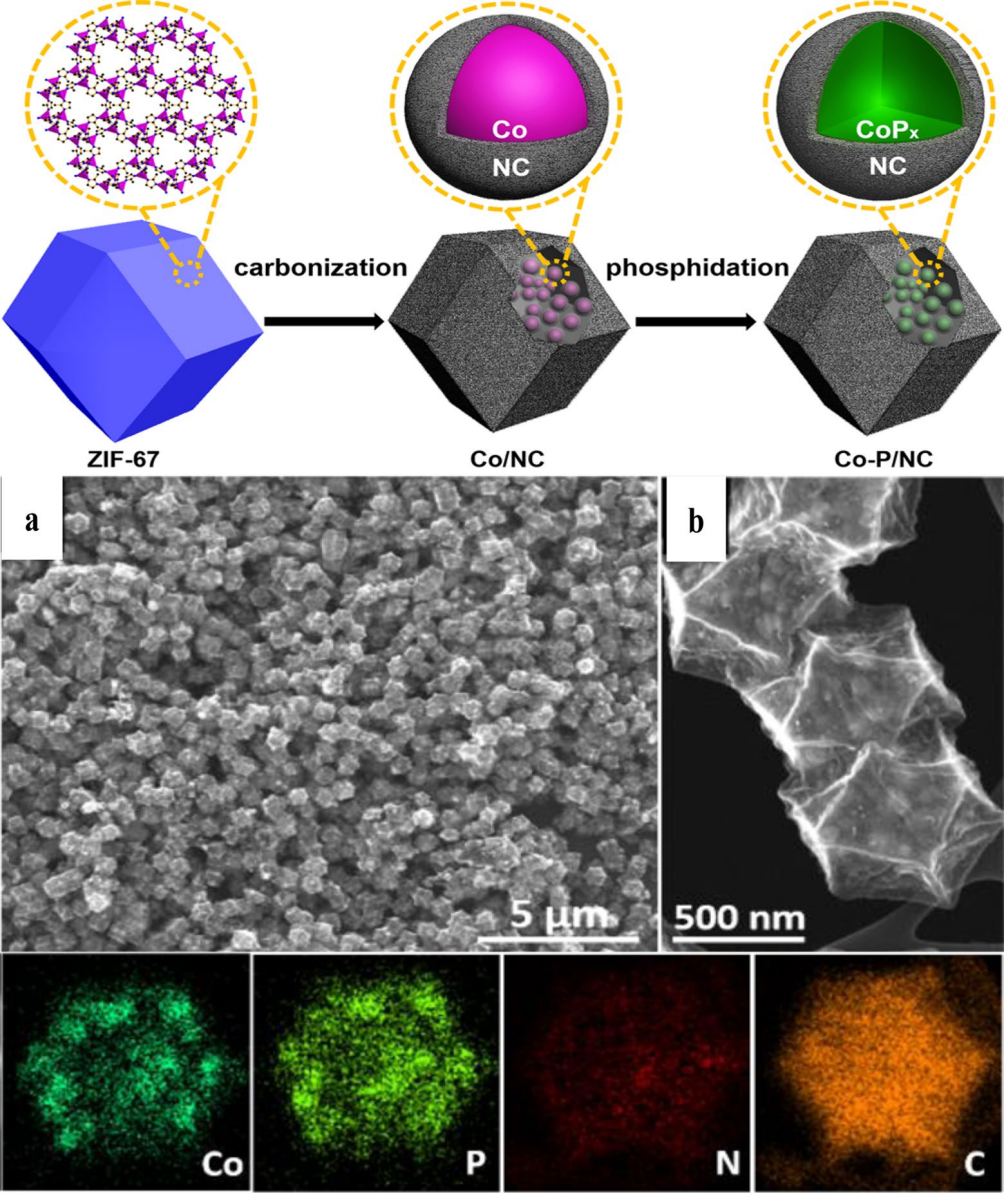
Schematic representation of the fabrication of Co-P/NC nanopolyhedrons. **a**, **b** are the SEM image and high magnification SEM image. The elemental mapping of Co-P/NC show the even elemental distributions of Co, P, N, and C. Reproduced with permission from Ref. [50]. Copyright 2015, American Chemical Society

He et al. have synthesized a multimetallic MOF, NiFe-MIL-53, and through a post-phosphorization step performed a replacement of bridging ligands toward efficient electrocatalysis [28]. In another important work by you et al., phosphorous was incorporated in a Co-based porous carbon-nitrogen framework using a simple physical mixing followed by a carbonization treatment. In this report, ZIF-67 was used as precursors to synthesize the Co-based high surface area porous carbon-nitrogen material, while phosphorization was performed in the second step (Fig. 4), where pre-synthesized carbonized MOF powder was mixed with a phosphorous source to obtain the final Co-P/NC compound [50].

**Fig. 5:**
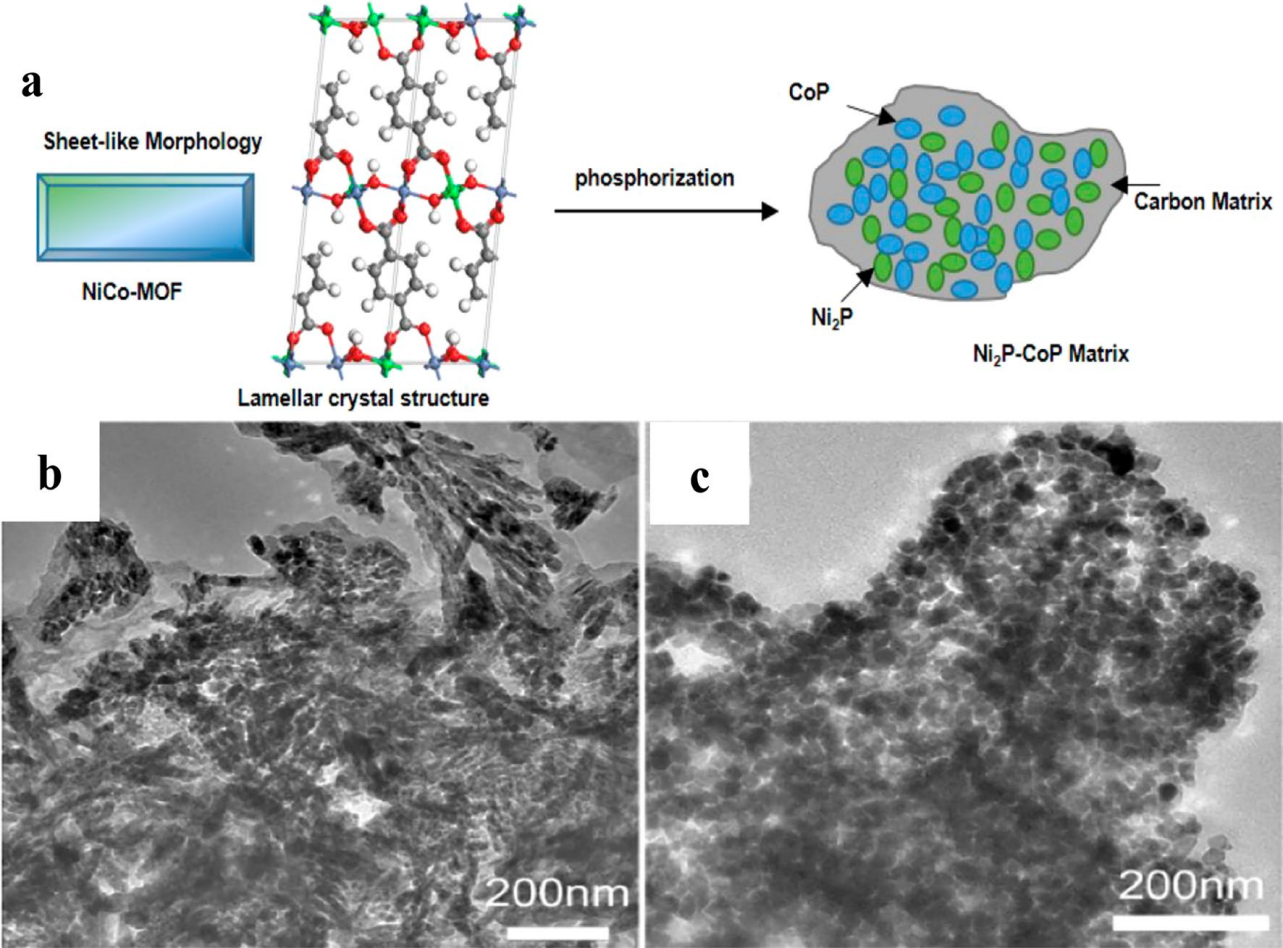
**a** Schematic representation of the fabrication of Ni_2_P − CoP matrix from NiCo-MOF. **b**, **c** TEM images of CoP and Ni_2_P nanoparticles, respectively. Reproduced with permission from Ref. [44]. Copyright 2015, American Chemical Society

Tang et al. have demonstrated the use of as-synthesized MoS_2_/CoS_2_ nanotube arrays as highly efficient catalysts by a multi-step fabrication process. At first, a Co-based MOF has been grown over carbon cloth electrodes. Thereafter, the MOF was subjected to an hydrothermal reaction to convert it into the final MoS_2_/CoS_2_ nanotube array structure [51]. Liang et al. shown phosphorization of bimetallic MOF to derive Ni_2_P-CoP nanostructure (As can be seen in the illustrative synthetic scheme and transmission electron microscopy (TEM) images in Fig. 5). In this work, the source of phosphorous was mixed with the MOF during the pyrolysis process. The above example shows the variety of nanomaterials, which can be synthesized using the carbonization of MOF with foreign doping of element during pre- and post- synthesis of MOFs [44]. These above-mentioned examples highlight the fact that MOFs provide an exceptionally versatile platform for the design and fabrication of functional carbon-metal nanostructures, having a wide-range of elemental compositions and chemical/physical properties.

## MOF-derived materials for electrocatalytic applications

MOF-derived material plays a key role in the design and development of efficient, potent electrocatalysts possessing unique attributes such as selectivity for the desired product, high density of solution-accessible catalytically-active sites, and stability under the harsh operational condition of electrocatalysis. In the past years, MOF-derived materials have demonstrated great potential to replace noble-metal based catalysts for a wide range of electrocatalytic energy conversion reactions as OER, ORR, HER, and many other oxidation processes, as vastly reported in literature [5, 52, 53]. Moreover, the alarming rise in global CO_2_ emission to the atmosphere demands for rapid development of advanced technologies enabling the production of clean, sustainable alternative fuels, replacing the traditional, polluting fossil fuels [52, 54]. In that regard, electrocatalytic reduction of CO_2_ is a promising solution, as it can lower the concentration of CO_2_ from the atmosphere and also produces valuable molecules, which can be further reused. Furthermore, yet another energy-consuming catalytic reaction is the industrial high-temperature synthesis of ammonia from N_2_ (Haber-Bosch process, which accounts for about 2% of the world’s energy demand) [55–60]. Thus, there is an urgent need to develop new efficient catalysts for both CO_2_ reduction reaction (CO_2_RR) and N_2_ reduction reaction (NRR), as will be further discussed over the next section.

## Electrochemical CO_2_ reduction reaction (CO_2_RR)

Electrochemical conversion of CO_2_ into alternative fuels and value-added chemicals is a promising approach to tackle the current energy demand and to reduce the concentration of CO_2_ in the atmosphere. Electrochemical CO_2_RR is a very intricate reaction, involving multi-electron and multi-proton transfer process to generate various types of surface reactions and reactive intermediates [54, 61]. In general, electrocatalytic CO_2_RR mainly encompasses three key steps: (1) the flux of CO_2_ toward the catalytic sites; (2) the surface reaction between the catalyst and CO_2_ to produce an adsorbed, stabilized catalytic-intermediate (usually through a 1-electron 1-proton transfer); (3) the desorption of the final product. Depending on the reaction’s pathway, a wide variety of carbon-based products are possible from C1 (i.e., one carbon atom containing) to C3 (i.e., three carbon atoms containing) products [54, 62–64]. To date, several ways have been attempted in order to prepare an high-performance MOF-derived CO_2_RR electrocatalyst, including: (i) generation of highly-porous structures to increase the density of active sites and promote efficient mass-transport within the catalysts pores, (ii) improving electrical conductivity by gaining control of the degree of graphitization, as well as ii) enhancing the intrinsic catalytic activity by fabrication of single-atom-based electrocatalysts [65, 66].

Commonly, MOF-derived materials based on ZIFs, tend to exhibit good CO_2_RR selectivity toward CO production, while Cu-based MOF-derived nanomaterials are intrinsically more selective for a multi-carbon product [61, 63] Table 1 provides a examples of state-of-the-art MOF-derived CO_2_RR electrocatalysts, with details regarding their preparation methods and catalytic performance characteristics.

Nam et al. have shown that a Cu-coordinated Cu-Cu CN framework could be synthesized from an HKUST-1 precursor, thus enabling selective CO_2_RR toward multi-carbon products [67]. Zhao et al. produced nitrogen-doped carbons from ZIF-8 to stabilize Ni single atom active-sites for selective CO formation with high turnover rates [65]. Additional evidence for the important role played by MOF-derived single atom based catalysts was provided by Wang et al. which used ZIF-67 to stabilize a single Co-atom catalyst for selective CO_2_ to CO formation [68]. In another work, a Fe-doped ZIF-8-derived material supported on carbon nanotube substrate, providing a conductive pathway toward the Fe-N active sites (embedded in the N-doped carbon derived from ZIF-8, via a two-step modification method that involves chemical modification followed by pyrolysis), and hence facilitating the production of CO with 100% efficiency [69]. Interestingly, application of MOF-derived aerogels were explored by Albo et al. for ethanol/methanol production, albeit with a very low faradaic efficiency of 10% [61]. In another report, Cardoso et al.. deposited ZIF-8 nanoparticles on Ti/TiO_2_ nanotubes for CO_2_RR, with low yield of ethanol generation [70]. Nevertheless, a Ru-doped Cu-based HKUST-1 (synthesized via an hydrothermal process), successfully provided product mixtures of ethanol/methanol during CO_2_RR, while exhibiting high faradaic efficiency of 47% [66]. Yet, despite the recent advance in the synthesis and application of MOF-derived materials for CO_2_RR, it appears that this field is still in his infancy, and there is much to be further explored in order to understand the fundamental principles controlling the activity and selectivity of these systems.

**Table 1:** Electrocatalytic CO_2_RR reduction using MOF-derived materials

Synthetic method	Electrode	Potential	Electrolyte	Product	FE (%)	Refs.
Pyrolysis	HKUST-1 derived Cu/carbon	− 0.7 V vs. RHE	1 M KOH	CO	71	[67]
Pyrolysis	ZIF derived Co-N	− 0.68 V vs. RHE	0.5 M KHCO_3_	CO	94	[68]
Pyrolysis	ZIF-8 derived NiSAs/N-C	− 0.89 V vs. RHE	0.5 M KHCO_3_	CO	72	[65]
Pyrolysis	HKUST-1 derived Cu-Cu CN	− 1.07 V vs. RHE	1 M KOH	C_2_H_4_	45	[67]
Pyrolysis	ZIF derived carbon and CNT	− 0.74 V vs. RHE	0.1 M NaHCO_3_	CO	97	[69]
Hydrothermal/pyrolysis	Ti/TiO_2_NT-ZIF-8	+ 0.1 V vs. RHE	0.1 M Na_2_SO_4_	C_2_H_5_OH	–	[70]
Hydrothermal	Cu based HKUST-1	− 1 V vs. Ag/AgCl	0.5 M KHCO_3_	CH_3_OH/C_2_H_5_OH	10.3	[61]
Hydrothermal	Ru doped HKUST-1	− 2 V vs. Ag/AgCl	0.5 M KHCO_3_	C_2_H_5_OH	47.2	[66]
Pyrolysis	ZIF-8 derived Fe-N sites	− 0.42 V vs. RHE	1 M KHCO_3_	CO	92	[64]

## Nitrogen reduction reaction (NRR)

Ammonia (NH_3_) is a valuable industrial feedstock-chemical, serving as an activated nitrogen source for fertilizers, plastics, textiles, and pharmaceuticals production. The traditional approach to produce NH_3_is by using the Haber-Bosch process, which requires substantial amount of energy and results in intensive CO_2_ emission. A promising, sustainable manner to produce NH_3_ is the electrochemical nitrogen reduction reaction (NRR) of using an efficient and robust electrocatalyst. NRR is considered a challenging task due to the difficulty of activating the inert N_2_ molecule, as a consequence of slow kinetics of N_2_ adsorption over heterogeneous surfaces while cleaving The stable nitrogen-nitrogen triple bond [59, 60]. Additionally, hydrogen evolution reaction (HER) constitutes a major competing side-reaction during NRR, frequently resulting in low reaction selectivity toward NH_3_ generation [55, 58, 60]. Hence. the key design rules for preparing an efficient NRR electrocatalyst are: (1) selection of suitable metal atom; (2) mitigation of the competing HER; (3) utilization of porous materials exposing high density of catalytically-active sites.

In that sense, MOF-derived porous materials with a suitable choice of metal atom could be a potent electrocatalyst for NRR. Indeed, there are several reports on MOF-derived NRR systems, as highlighted in Table 2. For instance, Mukherjee et al. have used an N-doped carbon derived from ZIF-8 as active NRR catalyst, possessing NH_3_ faradaic efficiency (FE) of 10% [41]. In another important work, ZIF-67 based MOF was used as a precursor to produce Co_3_O_4_@NCs core-shell structures via a two-step fabrication process of carbonization followed by oxidation in air. Interestingly, it was found that a synergetic effect between the N-doped carbon and Co_3_O_4_ accounts for the good NRR activity, resulting in FE of 8.5% [38]. Lu et al. fabricated Ni-based MOF-derived C@NiO@Ni microtubes that exhibited NH_3_ FE of 11% [71]. Wang et al. rationally designed an Fe-N/C NRR catalyst reaching NH3 FE of 9.2%, by carbonization of a Fe-ZIF-8-CNTs precursor at 1000 °C  [55]. In another work Lu et al. fabricated N-coordinated single Fe-sites by pyrolysing mixed-metal ZIF-8-MOF precursor at 1000 °C reported a high FE of 18.6% for N_2_ to NH_3_ conversion [72]. Overall, in spite of these recent examples, the amount of reports on MOF-derived electrocatalysts for NRR are still very limited. Hence, there is an obvious need to explore the vast chemical/structural versatility of MOFs in order to develop new, highly active and selective catalysts for electrochemical NH_3_ production.

**Table 2:** Electrocatalytic nitogen to ammonia conversion using MOF-derived material

Synthetic method	Electrode	Potential	Electrolyte	FE (%)	Refs.
Pyrolysis	Ni-BTC derived C@NiO@Ni	− 0.7 V vs. RHE	0.1 M KOH	11	[71]
Pyrolysis	ZIF-67 derived Co_3_O_4_@NC	− 0.2 V vs. RHE	0.05 M H_2_SO_4_	8.5	[38]
Pyrolysis	ZIF-8 derived Fe-N/C	− 0.2 V vs. RHE	0.1 M KOH	9.2	[56]
Pyrolysis	ZIF derived carbon	− 0.3 V vs. RHE	0.1 M KOH	10.2	[41]
Pyrolysis	ZIF derived Fe-N/C	− 0.4 V vs. RHE	0.1 M PBS	18.6	[72]

## Summary and future perspective

The continuously increasing number of reported MOFs structures has provided the opportunity to design new functional materials and to utilize them in energy-related electrochemical applications. Although direct use of pristine MOFs for electrocatalytic systems still suffers from relatively low conductivity, and lacking desirable chemical/structural robustness, MOF-derived materials are showing great promise for their use as catalysts, demonstrating exceptional electrochemical activity and stability. In particular, the notion of using MOFs as templated platforms to synthesize intricate nanostructured porous materials is of particularly high importance. These nanostructures may inherit essential structural and compositional properties from their MOF precursors, that may in turn be responsible for their remarkable electrochemical performance.

Nevertheless, in spite of the growing number of examples for using MOF-derived materials as electrocatalysts, further leap in their catalytic performance is still needed inorder to advance the field toward practical realization. Specifically, there are several key obstacles and drawbacks that still need to be overcomed: (i) In order to be able to design highly-efficient MOF-derived electrocatalysts, one should gain the ability to achieve fine-control over the structure, morphology, and elemental composition of the final derived materials. Yet, despite the great progress in the field, our knowledge of the precise MOF-conversion mechanisms is still very much limited. Hence, Future efforts should focus on obtaining new insights on the fundamental principles governing MOF-conversion, while understanding how to control and manipulate the chemical and structural properties of these MOF-derived compounds. Furthermore, there is a need t develop new means for MOF-conversion at milder condition than the traditional high-temperature pyrolysis. In that manner, it may be possible to obtain fine-control over the resulting material’s properties. (ii) Generally, in order to further improve the electrocatalytic performance of these MOF-derived materials, attention must be given to elucidate the relation between electrocatalyst’s structure and activity. Meaning, there is a need to gain deeper understanding of the catalyst’s active-site identity, while unveiling the details of the catalytic reaction mechanisms. By doing so, one can obtain data on the catalyst’s intrinsic activity, and design new strategies to construct more efficient MOF-derived electrocatalytic systems. We believe that a smart combination of computational and experimental efforts could pave the way for finding new, promising MOF-derived structures that will be able to overcome the abovementioned challenges. Lastly, we are certain that the important field of MOF-derived materials will continue to expand and involve contributions from multidisciplinary research areas over the next few years, and will lead to breakthroughs in a wide range of energy-related electrochemical reactions.

## Data Availability

Not applicable.

## Abbreviations

MOF: Metal–organic framework
OER: Oxygen evolution reaction
ORR: Oxygen reduction reaction
HER: Hydrogen evolution reaction
CO_2_RR: CO_2_ reduction reaction
NRR: N_2_ reduction reaction
